# Summer dry-down modulates the isotopic composition of soil CO_2_ production in snow-dominated landscapes

**DOI:** 10.1371/journal.pone.0197471

**Published:** 2018-05-10

**Authors:** Diego A. Riveros-Iregui, Theresa M. Lorenzo, Liyin L. Liang, Jia Hu

**Affiliations:** 1 Department of Geography, University of North Carolina at Chapel Hill, Chapel Hill, North Carolina, United States of America; 2 School of Life Sciences, Arizona State University, Tempe, Arizona, United States of America; 3 School of Science and Environmental Research Institute, University of Waikato, Hamilton, New Zealand; 4 School of Natural Resources and the Environment, University of Arizona, Tucson, Arizona, United States of America; University of Copenhagen, DENMARK

## Abstract

In mountainous landscapes, soil moisture is highly dynamic due to the effects of topography and the temporal variability imposed by seasonal precipitation, including rainfall and snow. Soil moisture is known to affect ecosystem carbon exchange both aboveground and belowground, as well as the stable isotopic composition of exchanged CO_2_. In this study we used an extensive suite of measurements to examine the effects of seasonal changes in soil moisture on the isotopic composition of soil CO_2_ production at the landscape level. We show that the seasonal decline in soil moisture (i.e., summer dry-down) appeared to impose a trend in the δ^13^C of soil CO_2_ production (δ_P_) with more negative δ_P_ early in the growing season when soils were wet, and more positive δ_P_ as the growing season progressed and soils dried out. This seemingly generalizable pattern for a snow-dominated watershed is likely to represent the variability of recently assimilated C, tracked through the plant-soil system and imprinted in the respired CO_2_. Thus, our observations suggest that, at least for mountainous environments, seasonal changes in δ_P_ are largely mediated by soil moisture and their spatial variability is partially organized by topography.

## Introduction

The hydrological cycle plays a large role in the expression of terrestrial ecosystem processes. Climate change is expected to intensify the hydrologic cycle in several ways, including by shifting the timing of springtime snowmelt towards earlier in the year in snow-dominated regions [1]. This phenomenon has been amply observed [2] and is expected to continue [3, 4] throughout the western United States. The timing and magnitude of snowmelt is not only a function of latitude, but is also strongly dependent on the physiographic characteristics of individual catchments [5]. While peak streamflows may increase along riparian corridors early in the growing season, high and steep areas of mountainous watersheds—which comprise the greatest areal extension of mountainous ecosystems—will dry out faster [6].

Reduction in water availability in sloping terrain can be problematic because it may translate into changes in ecosystem function at multiple levels. For example, reduced soil moisture in snow-dominated ecosystems is known to affect ecosystem carbon exchange both aboveground [7, 8] and belowground [9, 10]. Quantifying such changes is especially challenging for belowground processes, as factors driving changes in soil CO_2_ production and flux are complex and often confounding [11, 12]. Stable carbon isotope analysis is a useful tool to examine the response of soil CO_2_ production to changes in soil moisture and other environmental conditions [13–16]. Isotopic discrimination in plants as a result of photosynthesis and post-photosynthetic transport is reflected in the stable carbon isotopic composition of soil [17]. Similarly, the isotopic composition of soil CO_2_ is affected through the preferential diffusion of ^12^C-CO_2_ within the soil column as well as diffusion of atmospheric CO_2_ into the soil [18]. Thus, changes in soil moisture affect both the physical and biological processes that mediate the stable carbon isotopic composition of soil CO_2_ production (δ_P_).

Given the highly dynamic nature of soil moisture across mountainous landscapes [19], and considering further projections driven by climate change, we sought to evaluate the effects that the seasonal variability in soil moisture impose on the δ^13^C of soil CO_2_ production (δ_P_) at the landscape level. Specifically, we focused on the summer dry-down that forested ecosystems experience across the western U.S. In a related study, Liang et al. [20] reported that the spatial variability of soil moisture was a strong predictor of the δ^13^C of soil CO_2_ production in a subalpine forest; however, this study focused on comparing differences in soil moisture imposed by landscape position (riparian versus upslope), and did not examine the seasonal (i.e., temporal) dynamics in δ_P_ due to soil dry-down. Nonetheless, the findings confirmed direct measurements of the physiological response of plants to spatial differences in soil moisture as observed in the foliar ^13^C composition [21] and tree growth rates [22, 23]. The limitations of this study, however, were that the direct observations were conducted in a period of little temporal variability in soil moisture (i.e., late summer) and thus the effects of temporal effects of soil moisture on the isotope content of soil CO_2_ were not observed. Here, we describe how temporal changes in soil moisture may affect δ_P_ at the landscape level using extensive spatial coverage of the observations. We focus on observations collected over nine weeks in a subalpine watershed of the northern Rocky Mountains. This is a considerable advancement from previous studies because, to the best of our knowledge, this is the first study to examine the seasonal effects of soil moisture on spatially-distributed measurements of δ_P_, from the period immediately after snowmelt through late summer. We hypothesize that the soil moisture dry-down introduces a systematic change in δ_P_ and this can be observed at the landscape level. This study explicitly tests the role of topography and variable soil moisture on the isotopic composition of soil CO_2_ production measured at the landscape scale. Information gained through this research will aid in the interpretation of measurements from conventional eddy flux towers (which cannot resolve the spatial variability of soil or ecosystem CO_2_ fluxes across different landscape positions e.g., [24]), and contribute toward an improved mechanistic representation of terrestrial ecosystem carbon exchange in snow-dominated areas.

## Methods

### Site location

The study site was Stringer Creek, a subalpine watershed within the Tenderfoot Creek Experimental Forest (TCEF) in the Little Belt Mountains of Central Montana, United States. The U.S. Forest Service, Rocky Mountain Research Station (USFS-RMRS), which granted access to the site for this particular project, operates TCEF. The greater TCEF has an area of 3,591 ha, an elevation range of 1,838 to 2,421 m (average is 2,206 m), and receives an average 890 mm of precipitation annually, ranging from 595m at lower elevations to 1050mm at its upper slopes [25]. Snow reaches its maximum rate of precipitation during the months of December and January and accounts for over 70% of total annual precipitation [26]. Peak streamflow is snowmelt-driven and is usually observed in early June [27]. Optimal soil water content for soil CO_2_ efflux has been found between 40% and 60% following snowmelt [28], with highest soil CO_2_ concentrations under 20 cm. The vegetation of this site consists of C_3_ plants, including lodgepole pine (*Pinus contorta)*, Engelmann spruce (*Picea eglemanni*), whitebark pine (*Pinus albicaulis*), subalpine fir (*Abies lasiocarpa*), and grouse whortleberry (*Vaccinium scoparium*) in upland areas, and blue joint reed grass (*Calamagrotis canadensis)* along riparian meadows. The most dominant soil groups are loamy, skeletal, mixed Typic Cryochrepts in upland areas and highly organic, clayey, mixed Aquic Cryoboralfs in the riparian areas [29]. Soil bulk density ranges from 0.962 g cm^-3^ in riparian meadows to 0.911 g cm^-3^ in uplands, with no significant differences in soil bulk density or root density reported [30].

### Field sampling

We report on measurements collected over nine weeks, from June 8 to August 9, 2013 at thirty-two soil plots located in Stringer Creek, a sub-watershed of TCEF. Plots were distributed throughout the 393-ha watershed with 23 of those in upland forests and 9 along riparian meadows. Soil CO_2_ was collected from existing gas wells installed at depths of 5, 20, and 50 cm in each plot and described in detail by a previous study [28]. The gas wells consisted of 15-cm PVC sections of 5.25 inside diameter capped with rubber stoppers (size 11), inserted into augered holes and open at the depth of interest to allow for gas equilibrium between the gas well and the surrounding soil. PVC tubing (4.8mm inside diameter Nalgene 180 clear PVC, Nalge Nunc International, Rochester, N.Y., USA) extended from the gas wells to the ground surface. The PVC tubing was joined with connectors above the surface to prevent leaks from the gas wells between measurements. A total of 96 gas wells were used in this study (i.e., 32 plots × 3 depths).

The air space within each gas well was sampled at sub-weekly time intervals using a handheld carbon dioxide analyzer equipped with a pump (GM-70, Vaisala GM70, Woburn, MA), connected in line with a sampling valve. During gas sampling, air was circulated and monitored using the GM-70 to ensure that no sudden changes in CO_2_ concentration occurred inside the gas well, an indication of leaks in the system. Soil gas samples were taken usually 3–4 days apart, allowing enough time for the soil gas atmosphere to equilibrate. From each gas well, 120 ml of soil gas was extracted and injected into a 180-ml Tedlar sample bag (SKC Inc., Eighty Four, PA) that had been previously flushed with N_2_. Simultaneously, we measured volumetric water content of the soil at a depth of 0–12 cm (HydroSense, Campbell Scientific, UT), soil temperature (12 cm soil thermometer, Reotemp Instrument Corporation, San Diego, CA), and soil CO_2_ efflux (EGM-4 with SRC-1 respiration chamber, PP Systems, Amesbury, MA) three times per plot. Within 4 hr of collection, Tedlar bags were connected to a Cavity Ring-Down Spectroscopy (CRDS) analyzer (model Picarro G2101-*i*, Picarro Inc., Sunnyvale, California) to measure the δ^13^C composition of soil CO_2_ in the Tedlar bag. Two standard gases were used to calibrate the performance of the CRDS analyzer daily. Routine checks using these standards, instrumentation, and sampling setup yielded a repeatability of the instrument better than 0.2‰. All isotope values are reported using the delta notation and relative to the international standard of Vienna Pee Dee Belemnite (VPDB). As most groups evaluated exhibited non-normal distributions and unequal variances, we used Welch’s one-way ANOVA for group comparison [31].

### Isotopic mixing relationships

Under steady state conditions, the δ^13^C of soil CO_2_ flux is equivalent to the δ^13^C of soil CO_2_ production [32]. We applied the Keeling plot method [33] using the measured CO_2_ concentration and its stable isotopic composition from each depth. Thus, a Keeling plot consisted of an ordinary least-squares regression between the observed carbon isotopic compositions of the four samples (i.e., 5, 20, and 50 cm plus above air) and the inverse CO_2_ concentrations (i.e., 1/[CO_2_]) of the same four points. We used time- and site-specific measurements of above air (air above each plot at the time of sample collection). δ_P_ was calculated by subtracting 4.4‰ (diffusive depletion constant [18]) from the estimated intercept of each ordinary least-squares regression. Any regression with a standard error of the intercept above 1‰ was discarded, so only the most robust regressions were used for further analysis [24]. Recent studies have shown that shallow soils are more prone to errors due to the proximity to the forest floor and the potential for artificial mixing with forest air via advection [16]. This was confirmed by our previous work, which showed that Keeling-plot derived δ_P_ is not overly sensitive to errors at any particular depth given that it is a depth-averaged technique, and thus it provides greater confidence in the isotopic composition of soil CO_2_ production, particularly for surface layers [20]. We performed the Wilcoxon rank sum test to evaluate statistical differences in soil moisture and δ_P_ across the growing season. SPSS 19 (IBM, Armonk, NY) was used to execute the Welch’s ANOVA. All other statistical analyses and data processing were performed using MATLAB R2017a (The MathWorks Inc., Natick, MA, USA).

## Results

**Fig 1:**
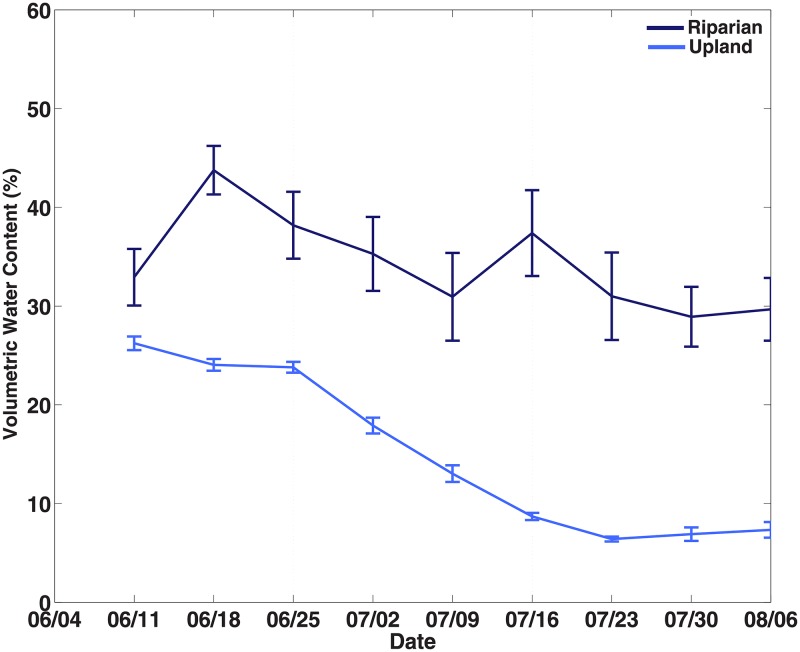
Seasonal soil moisture. Weekly averaged soil moisture in upland and riparian soil plots during the 2013 growing season.

Weekly averages of soil moisture are shown in Fig 1, separated into upland and riparian soil plots. Throughout the growing season, there was a clear decrease in soil water content across the landscape, although such a decrease is more pronounced in uplands areas where soil moisture ranges from ~25% on average in early June to less than 10% in early August. In contrast, the soil moisture in riparian areas decreased much more slowly, only reaching below 30% in the last three weeks. Given that our overarching goal was to evaluate the effects of seasonal changes in soil moisture, we used the dynamics of measured soil moisture to divide the growing season into three distinct periods: a wet period (Weeks 1–3), characterized by a slow decrease in soil moisture; a transition period (Weeks 4–6), characterized by the fastest rate of decrease in soil moisture of the growing season; and a dry period (Weeks 7–9), characterized by dry soils with little change in soil moisture. These periods and dynamics were especially evident across all upland plots (Fig 1), where these differences in soil moisture were significant across the three periods (p<0.0001 in all cases). No significant differences were found for riparian locations. Given that upland locations represent 98% of the watershed area for this catchment [10], the analysis below refers to these three periods.

**Fig 2:**
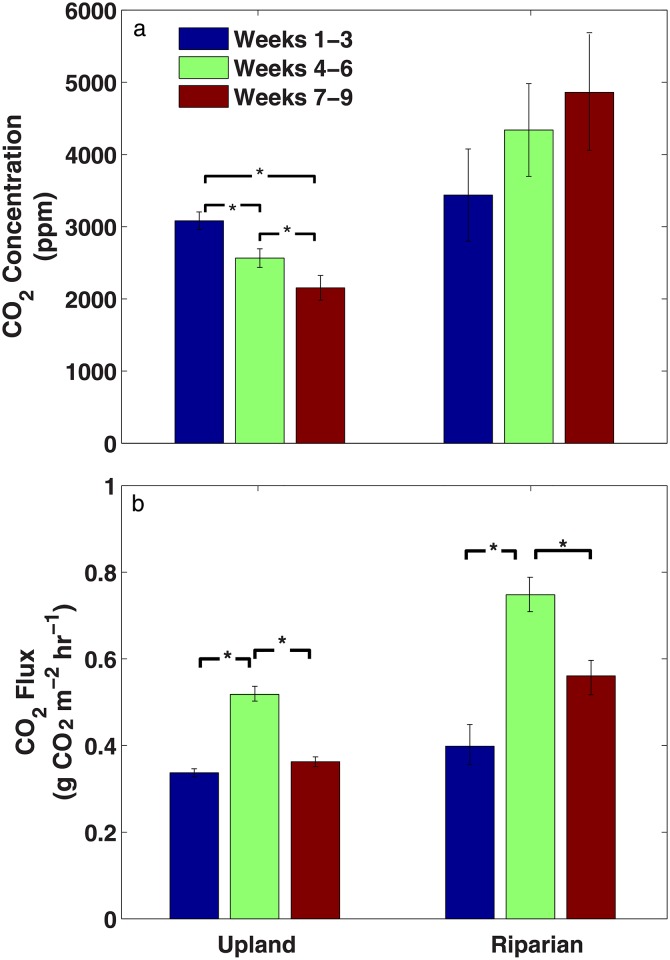
Soil CO_2_ concentrations and flux. Comparison of mean CO_2_ concentrations across three depths and flux between and within upland (n = 23) and riparian (n = 9) plots during wet (Weeks 1–3), transition (Weeks 4–6), and dry (Weeks 7–9) periods of the 2013 growing season. Asterisks (*) indicate significant differences between groups (p<0.05, Welch’s ANOVA). Bars indicate standard errors of the mean.

Regarding soil CO_2_ concentrations, we found no significant differences between the CO_2_ concentrations between upland and riparian plots at the beginning of the growing season, although such differences did emerge as the season progressed as a result of CO_2_ concentrations systematically increasing in riparian plots and decreasing in upland plots (Fig 2A). On the other hand, soil CO_2_ flux was consistently higher in riparian plots than uplands, having its highest fluxes during the transition period (i.e., weeks 4–6) across all sites (Fig 2B).

**Fig 3:**
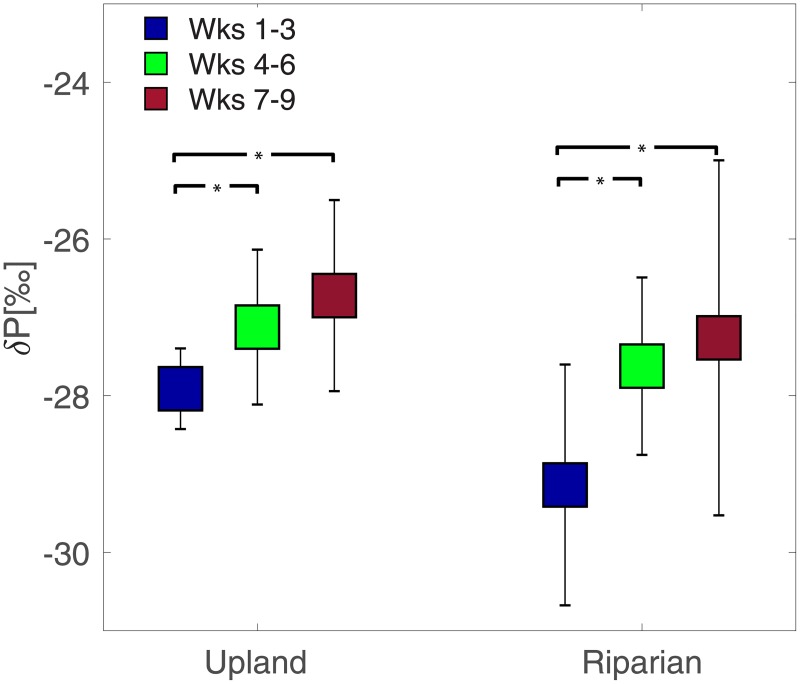
Seasonal evolution of δ_P_ across upland and riparian soil plots. Squares denote means across upland (n = 23) and riparian (n = 9) sites, whereas error bars denote standard deviations. Asterisks denote statistical differences between different sampling periods (p<0.05, Wilcoxon rank sum test).

**Fig 4:**
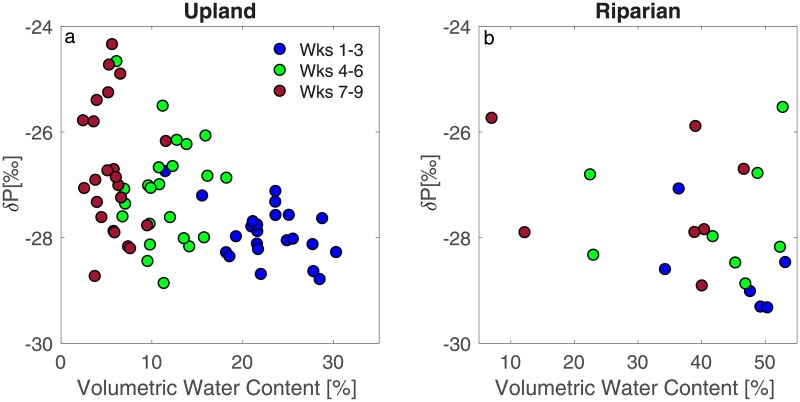
Volumetric water content and the δ^13^C of soil CO_2_ production (δ_P_). Volumetric water content was a significant predictor of the variability of δ_P_ across upland (R^2^ = 0.23, p<0.0001) and riparian plots (R^2^ = 0.18, p<0.05). Note x-axis range is different in each panel.

Across both landscape positions, δ_P_ was more negative during the wet period and became progressively more positive toward the transition and dry period (Fig 3). It is worth noting that these differences were statistically significant between the wet period and each of the subsequent two periods, but the differences were not statistically significant between the transition and dry periods (Fig 3), likely as a result of the increased variability among measured plots. Compared to the measured volumetric water content, the measured δ_P_ showed variable dynamics in upland plots vs. riparian plots at different times of the growing season (Fig 4). Soil moisture was a significant predictor of the variability of δ_P_ across riparian (R^2^ = 0.18, p<0.05) and upland plots (R^2^ = 0.23, p<0.0001) combined, and only during the wet period for upland plots (R^2^ = 0.21, p<0.05), likely as a result of a narrower range in soil moisture during the other periods (i.e., transition and dry periods). In the uplands, the range of δ_P_ (minimum – maximum value) was around ~2‰ early in the growing season when soils were wetter, whereas the same range increases to over 5‰ when soils were drier. In riparian areas these differences were not as clear.

## Discussion

Our experimental design took advantage of the long and pronounced summer dry-down following snowmelt at a site where snow contributes most of the water for these mountainous ecosystems [27], and of the monotonic decline in soil moisture throughout the growing season, especially evident in sloping areas (Fig 1). Of particular note in our results was: 1) soil CO_2_ concentrations increased in riparian plots as the growing season progressed, whereas the same concentrations systematically decreased in uplands; 2) the peak in soil CO_2_ flux occurred toward the middle of the growing season for both riparian and upland plots; and 3) the seasonal decline in soil moisture (i.e., summer dry-down) appeared to impose a trend in δ_P_ with more depleted δ_P_ early in the growing season when soils were wet, and more positive δ_P_ as the growing season progressed and soils dried out.

Plot-level observations showed that soil moisture in well-drained upland areas was significantly lower than soil moisture in riparian areas (Fig 1; one-way ANOVA, p<0.01), which can be attributed to the higher topographical position combined with the shallow, coarser soil texture relative to the poorly drained soil found in riparian plots. Across all locations, soil moisture was strongly, inversely correlated with plot elevation (r = -0.33; p<0.0001), a variable that is known to integrate important topographic characteristics such as porosity, hydraulic conductivity, aspect, clay content, specific contributing area, wetness index, curvature, and soil depth [34, 35]. And while the seasonal dry-down in soil moisture is evident across an entire growing season, it is not as evident within single 3-week periods, likely because not all sites dry down equally and the local microtopography can play a big role in the short-term dynamics or soil moisture at the site level [36]. In addition, previous studies at this same research site have shown that wet landscape positions have higher fine root biomass and lower carbon-to-nitrogen (C:N) ratios than dry landscape positions, likely as a result of differences in vegetative cover (grasses vs. trees) [10]. Low C:N ratios are known to contribute to rapid litter decomposition rates [37], whereas high root biomass can contribute to high generation and flux of soil CO_2_ [38]. Both models and experimental studies have shown that soil moisture generally enhances both soil CO_2_ concentration and CO_2_ flux, although at very high soil moisture levels both gas diffusivity and production are inhibited [39–42]. Our results also suggest that for this mountainous ecosystem, soil CO_2_ flux is both diffusion-limited and production-limited (Fig 2). Early in the growing season during the wet period (Weeks 1–3), soil CO_2_ flux appeared to be diffusion-limited despite soil CO_2_ concentrations being slightly above 3000 ppm on average (Fig 2). Later, during the dry period (Weeks 7–9), soil CO_2_ flux was only slightly higher than during the wet period but significantly lower than during the transition period (Weeks 4–6) in response to soil CO_2_ concentrations that have increased to near 5000 ppm in riparian areas and decreased to around 2000 ppm in the uplands. In addition, it appeared that even by late summer, riparian plots remained diffusion-limited whereas upland plots were production-limited (Fig 2). These low-high-low patterns in soil CO_2_ flux (Fig 2B) have been previously attributed to the stimulation by soil moisture of heterotrophic and autotrophic soil CO_2_ production [39, 43], combined with the limitation that soil moisture imposes on soil gas transport [44, 45]. Our results appear to suggest the emergence and spatial manifestation of this well-established parabolic relationship across the landscape.

A previous study using three soil plots in a maritime pine forest [46] showed that the δ^13^C of soil CO_2_ flux signal became relatively more positive as the growing season progressed, coinciding with the maximum rates of soil CO_2_ flux. However, the same study showed greater variability after summer rainfall arrived, which was attributed to differences in the isotopic signatures of heterotrophic and autotrophic respirations and changes in their relative contribution. Separating the contribution of δ^13^C from heterotrophic respiration versus autotrophic respiration is notoriously difficult. Hanson et al. [47] reviewed over 50 published studies that used a variety of methods to partition heterotrophic versus autotrophic respiration and found that on average, root respiration contributed to 48% of total soil respiration, with this value slightly higher for forest soils. As for the heterotrophic respiration, studies show a positive relationship between increasing soil moisture and heterotrophic respiration rates [48], and since fractionation is assumed to be negligible during respiration [49] heterotrophic respired δ^13^C will have a similar signature to plant-derived tissue. Thus, while we recognize that heterotrophic respired δ^13^C may be an important and un-accounted source of CO_2_ to the total CO_2_ flux pool, the systematic increase in δ_P_ derived from analysis of 23 upland and 9 riparian plots, appears to be a generalizable pattern for this snow-dominated watershed (~4 km^2^ in area; Fig 3).

In water-limited ecosystems, studies have demonstrated that δ^13^C of whole ecosystem respiration tends to become more positive as soil moisture decreases throughout a season [24, 50] or across sites ranging in annual precipitation inputs [51, 52]. The reason is that soil moisture influences photosynthetic discrimination by C_3_ plants through stomatal conductance, as proposed by Farquhar et al. [53, 54]: as plants experience water limitation and stomatal conductance decreases, less discrimination against ^13^C by Rubisco results in more positive δ^13^C in the recently fixed assimilations. These enriched assimilates are then transported to other parts of the plant, where this carbon source can be used for respiration. Studies have shown that because these recently fixed sugars are transferred to the roots, δ^13^C of root respiration can reflect δ^13^C of photosynthates over relatively short time periods [13, 55]. In our study, the increase in δ_P_ with decreasing soil moisture across the growing season (Fig 4) follows this line of reasoning. Because C transport in the plant itself is not assumed to fractionate against ^13^C [56], it is likely that the observed seasonal pattern is the result of the effects of soil moisture on recently assimilated C, tracked through the plant-soil system and imprinted in the respired CO_2_.

Previous studies have tracked the δ^13^C of soil respired CO_2_ in C_3_ plants in response to day-to-day variation in the photosynthetic carbon isotope fractionation [13, 57–60], suggesting that half or more of the biological activity in the soil is driven by recent tree photosynthate. Atmospheric vapor pressure and precipitation have been reported as drivers of the δ^13^C of ecosystem respired CO_2_ [15, 51]. More recent studies, however, have found that relationships with atmospheric vapor pressure and precipitation are not consistent across the growing season at the ecosystem [24, 61] or even the soil level [46], making it not possible to directly relate the δ^13^C to plant assimilated carbon to potential variations in stomatal conductance. On the other hand, soil moisture exerts a direct control on the resulting δ^13^C of soil- or ecosystem-respired CO_2_ [20, 62–65], but summer-long patterns have been difficult to identify [24, 46], in part because the confounding effects of the multiple variables that regulate both soil moisture and leaf carbon exchange. To the best of our knowledge, this is the first study to report a generalizable pattern in the δ^13^C of soil CO_2_ production (δ_P_) as a result of the summer dry-down of soil moisture using multiple locations in a mountainous watershed.

Soil moisture is an important factor in ecosystem CO_2_ exchange through its influence on physiological and soil physical processes. Our study suggests that in mountainous environments with high to medium soil water content, the summer soil moisture dry-down modulates the δ^13^C of soil CO_2_ production. We showed that the seasonal decline in soil moisture (i.e., summer dry-down) appeared to impose a trend in the δ^13^C of soil CO_2_ production (δ_P_) with more negative δ_P_ early in the growing season when soils were wet, and more positive δ_P_ as the growing season progressed and soils dried out. This seemingly generalizable pattern for a snow-dominated watershed is likely to represent the variability of recently assimilated C in the plant-soil-atmosphere continuum. Our observations suggest that, at least for mountainous environments, seasonal changes in δ_P_ are largely mediated by soil moisture and their spatial variability is partially organized by topography. Additional research is needed to further establish the role of stochastic precipitation on masking this relationship, as well as the role of variable soil moisture on the separate isotopic contributions from heterotrophic and autotrophic respiration.

Isotopic mass balance at the ecosystem level is currently unachievable because of large gaps in our understanding of several processes, including the dynamics of leaf, plant, and soil processes in response to environmental conditions [66]. Climate models project an increasing probability of water stress on soil and plant communities throughout the western United States due to reduction of snow accumulation. Changes in water availability at the landscape level would not only affect the rate of soil carbon turnover, but it would also affect the isotopic composition of soil CO_2_ production and presumably whole ecosystem respiration. Our study provides evidence to suggest that the parameterization of soil moisture heterogeneity in space and time is a required component of realistic representations carbon isotope exchange in mountainous regions.

## Supporting information

S1 Table Dataset used in this manuscript. Sites T1W1, T1W2, T1E1, T1E2, T2W1, T2W2, T2E1, and T2E2 are located in riparian areas. Sites T1W3, T1W4, T1E3, T1E4, NW-, and SW- are located in upland areas.
(XLSX)
